# Nutritional Analysis of Foods and Beverages Posted in Social Media Accounts of Highly Followed Celebrities

**DOI:** 10.1001/jamanetworkopen.2021.43087

**Published:** 2022-01-12

**Authors:** Bradley P. Turnwald, Kathryn G. Anderson, Hazel Rose Markus, Alia J. Crum

**Affiliations:** 1 Booth School of Business, University of Chicago, Chicago, Illinois; 2 Department of Psychology, Stanford University, Stanford, California

## Abstract

**Importance:**

Celebrity social media posts engage millions of young followers daily, but the nutritional quality of foods and beverages in such posts, sponsored and unsponsored, is unknown.

**Objective:**

To quantify the nutritional quality of foods and beverages depicted in social media accounts of highly followed celebrities and assess whether nutritional quality is associated with post sponsorship, celebrity profession or gender, and followers’ likes and comments.

**Design, Setting, and Participants:**

This cross-sectional study analyzed the content of food- and beverage-containing posts from Instagram (a photo- and video-sharing social media platform) accounts of 181 highly followed athletes, actors, actresses, television personalities, and music artists. Data were collected from May 2019 to March 2020.

**Main Outcomes and Measures:**

The nutritional quality of foods and beverages posted in celebrity social media accounts was rated using the Nutrient Profile Index (NPI) based on the sugar, sodium, energy, saturated fat, fiber, protein, and fruit and/or vegetable content per 100-g sample (a score of 0 indicated least healthy and 100, healthiest); foods with scores less than 64 and beverages with scores less than 70 were rated as “less healthy.” Secondary outcomes were whether the nutritional quality of foods and beverages in social media posts was associated with post sponsorship, celebrity profession or gender, and followers’ likes and comments. Mixed-effects regression models were used to estimate how outcomes differed across fixed effects.

**Results:**

The sample included social media accounts of 181 celebrities (66 actors, actresses, and television personalities [36.5%]; 64 music artists [35.4%]; and 51 athletes [28.2%]). A total of 102 celebrities (56.4%) were male, and the median age was 32 years (range, 17-73 years). Among 3065 social media posts containing 5180 total foods and beverages (2467 foods [47.6%]; 2713 beverages [52.4%]), snacks and sweets (920 [37.3%] of the foods) and alcoholic beverages (1375 [50.7%] of the beverages) were most common. Overall, 158 celebrity social media accounts (87.3%) earned a less healthy overall food nutrition score and 162 (89.5%) earned a less healthy overall beverage nutrition score, which would be unhealthy enough to fail legal youth advertising limits in the UK. For foods, social media posts with healthier nutrition scores were associated with significantly fewer likes (*b*, –0.003; 95% CI, –0.006 to 0.000; *P* = .04) and comments (*b*, –0.006; 95% CI, –0.009 to –0.003; *P* < .001) from followers. For beverages, nutrition scores were not significantly associated with likes (*b*, –0.010; 95% CI, –0.025 to 0.005; *P* = .18) or comments (*b*, –0.003; 95% CI, –0.022 to 0.016; *P* = .73). Only 147 food- or beverage-containing posts (4.8%) were sponsored by food- or beverage-relevant companies. Beverages in sponsored posts contained more than twice as much alcohol as those in nonsponsored posts (10.8 g [95% CI, 9.3 g to 12.3 g] per 100 g of beverage vs 5.3 g [95% CI, 4.7 g to 5.9 g] per 100 g of beverage).

**Conclusions and Relevance:**

In this cross-sectional study, most highly followed celebrity social media accounts depicted an unhealthy profile of foods and beverages, primarily in nonsponsored posts. These results suggest that influential depictions of unhealthy food and beverage consumption on social media may be a sociocultural problem that extends beyond advertisements and sponsorships, reinforcing unhealthy consumption norms.

## Key Points

**Question:**

What is the nutritional quality of foods and beverages depicted by celebrities on social media platforms?

**Findings:**

In this cross-sectional study of 5180 foods and beverages in social media posts from 181 highly followed celebrities, more than 87% of the celebrity social media accounts had posts of foods and beverages with less healthy Nutrient Profile Index ratings, such as alcoholic beverages and snacks and sweets. Only 4.8% of food- and beverage-containing posts were sponsored by food or beverage companies.

**Meaning:**

The findings suggest that the greater proportion of highly followed celebrity social media accounts depict foods and beverages with an unhealthy profile, primarily in nonsponsored posts.

## Teaser

This cross-sectional study quantifies the nutritional quality of foods and beverages posted on social media accounts of highly followed celebrities and assesses the association of nutritional quality with post sponsorship, celebrity profession and sex, and followers’ likes and comments.

## Introduction

Worldwide, traditional advertisements feature unhealthy foods and often target youths.^1,2,3,4,5,6^ As a result, many countries have adopted measures to limit children’s and adolescents’ exposure to food marketing on television.^7,8^ However, youths have rapidly migrated from traditional media to social media such as Instagram and Twitter.^9,10^ Social media have greater influential potential than traditional media, allowing users to choose targeted content and to interact with posts through liking and commenting. Popular brands’ social media accounts have capitalized on these features to promote unhealthy foods and beverages to youths online.^11,12,13,14^

However, the influential power of social media expands beyond advertisements. Social media shape, maintain, and update users’ perceptions of social norms.^15,16,17,18^ Creating the perception that users are broadcasting their real life, social media platforms boost perceived authenticity and credibility of posted content.^19^ Celebrities are particularly influential.^20^ On social media, celebrities are perceived as fellow users but also as more credible than ordinary users and more trustworthy than television advertisements.^19,21,22^ Celebrity posts can influence viewers through attitude alignment,^23^ social connection,^24^ and positive meaning transfer from likeable people to the foods and beverages that they depict.^25^

Little is known about the foods and beverages posted on social media by popular actors and actresses, athletes, and music artists. Research describing foods and beverages on social media has focused primarily on brands’ accounts^11,12,13,26^ and influencers (everyday people who build fame from creating engaging social media content)^27,28,29,30^ rather than mainstream celebrities. It is well known that celebrities endorse unhealthy foods and beverages to youth viewership in traditional advertising.^31,32,33^ However, on social media, celebrities post a hybrid of sponsored posts and personal posts that feature foods and beverages as authentic aspects of daily life.^19^ Experiments suggest that both sponsored and nonsponsored food and beverage posts can influence viewers’ attitudes and behavior,^19,29,34^ but the full profile of foods and beverages posted by celebrities on social media is unknown.

To address these questions, the present study systematically quantified the nutritional quality of 5180 foods and beverages posted on Instagram (a photo- and video-sharing social media platform) by 181 highly followed athletes, actors, actresses, television personalities, and music artists. We focused on this platform because it is image-driven, with more than 500 million daily active users, including 72% of teens in the US, and a large celebrity presence.^35^ We used several nutrition scoring systems to test our primary hypothesis that the profile of foods and beverages depicted in celebrity social media accounts would be primarily unhealthy. Exploratory analyses further compared nutritional quality in sponsored vs nonsponsored posts, followers’ interactions with posts via likes and comments, and differences by celebrity profession and gender.

## Methods

### Celebrity Sample

This cross-sectional study followed the Strengthening the Reporting of Observational Studies in Epidemiology (STROBE) reporting guideline.^36^ We selected 200 of the most popular athletes, music artists, actors, actresses, and television personalities by consulting 4 lists: the ESPN 2018 World Fame 100,^37^ 2018 Billboard year-end top 100 artists,^38^ 2018 Internet Movie Database (IMDb) 100 most in-demand actors and actresses,^39^ and Trackalytics’ most followed profiles on the platform.^40^ The ESPN World Fame 100 list, billboard year-end lists, and IMDb lists have been used in prior research^41,42,43,44,45,46^ to identify influential celebrities, songs, or movies, and Trackalytics provides updated follower counts for highly followed social media accounts. To balance by gender, the top 25 females and top 25 males were selected from each list. If there were fewer than 25 females on a given list, additional males were chosen until 50 celebrities were selected. When a celebrity on the Trackalytics rankings overlapped with a celebrity from another list, the next celebrity was selected until 50 were reached from each list. The platform verifies the authenticity of celebrities’ accounts and displays a badge for verified accounts. Of the 200 celebrities selected, 19 did not have a verified account on the platform and were excluded. This yielded a final sample of 181 celebrities (eTable 1 in the Supplement). The Stanford University institutional review board deemed this study exempt from the need for approval and informed consent because it did not qualify as human participants research. Posts on the platform were publicly available.

### Collecting Social Media Posts and Identifying Foods and Beverages

For each celebrity social media account, up to 30 of the most recent posts depicting a food or beverage were identified. Videos were excluded because viewers who do not watch an entire video would not see foods and beverages that appear partway through the video. Stories on the platform were also excluded because they disappear after 24 hours and were no longer visible during data collection. This resulted in a sample of 3065 social media posts (median, 20 [range, 1-30] per celebrity) posted from April 2012 to March 2020. Two celebrity accounts had no food or beverage posts. For each post, food and beverage items were coded as specifically as possible using the image and caption (eg, “Red Bull energy drink” instead of “energy drink”) (eTable 2 in the Supplement describes special cases). Food or beverage types appearing multiple times per post were coded only once. The numbers of likes and comments per post were recorded. Data were collected from May 2019 to March 2020.

### Sponsored Social Media Posts

We coded whether each post was explicitly disclosed as being sponsored by a food- or beverage-relevant company. Sponsored social media posts are paid advertisements, indicated with terms such as “paid advertisement,” “#advert,” “#ad,” or “sponsored” below the post. The Federal Trade Commission regulates social media sponsorship disclosures^47^ and has cited celebrities for not properly disclosing sponsored posts.^48^

### Obtaining Nutrition Information From US Government Databases

To obtain nutritional values, each unique food (n = 866) and beverage (n = 277) was matched to an entry in the US Department of Agriculture’s Food and Nutrient Database for Dietary Studies (FNDDS), 2015-2016.^49^ The FNDDS contains standard nutritional values per 100-g portion for more than 8600 foods and beverages. When specific details about a food were unclear from the social media post, the general FNDDS entry for that food type was selected (eg, “cheeseburger, not further specified”). For each food and beverage, we recorded the sugar, sodium, saturated fat, total fat, energy, protein, and fiber content from the closest matching FNDDS entry, which were required for nutrition rating calculations. For reporting food and beverage category frequencies, we used the US Department of Agriculture’s 2015-2016 What We Eat In America designations,^50^ which group foods into 11 categories (fruits, vegetables, dairy, proteins, grains, mixed dishes, snacks and sweets, fats and oils, condiments and sauces, sugars, and protein and nutritional powders) and beverages into 8 categories (alcoholic beverages, sweetened beverages, water, coffee and tea, dairy beverages, 100% juices, diet beverages, and infant formula and human milk), each with multiple subcategories.

### Monitoring Coder Reliability

As in prior research,^12,46,51^ interrater reliability between the 2 researchers who conducted the coding (B.P.T. and K.G.A.) was rigorously monitored at each step (eMethods in the Supplement). Interrater reliability was high for agreeing on the type of food or beverage depicted (κ range, 0.80-0.96), post sponsorship (96.7% agreement [κ, 0.82]), celebrity gender (100% agreement [κ, 1.00]), and FNDDS code (92.7%-96.4% agreement).^52^

### Classifying Nutritional Content

To evaluate nutritional quality, we used 2 established nutrition rating systems used in UK advertising law.^7,53,54,55^ First, as in prior research,^31,32,33,45,54^ we used the Nutrient Profile Index (NPI) to generate nutrition scores from 0 (least healthy) to 100 (healthiest) based on the sugar, sodium, energy, saturated fat, fiber, protein, and fruit and/or vegetable content per 100-g sample of the food or beverage. According to UK advertising guidelines, foods with NPI scores less than 64 and beverages with scores less than 70 are considered less healthy and are unlawful in traditional media advertisements to youths.^7^ Second, we used the front-of-package traffic light labeling guidelines^55^ to classify the sugar, saturated fat, total fat, and sodium content in foods as low (green traffic light), medium (amber traffic light), or high (red traffic light). Neither rating system depends on depicted portion sizes. They evaluate the nutritional quality of foods and beverages per 100-g sample, making them ideally suited for our research questions.

### Statistical Analysis

To estimate outcomes (eg, NPI scores) for each celebrity, we used mixed-effects regression models from the lmerTest package^56^ in R, version 3.6.2^57^ (R Project for Statistical Computing) that accounted for the nested random effects structure of foods and beverages nested within posts, nested within each celebrity. To estimate how outcomes differed across fixed effects (eg, post sponsorship), mixed-effects models estimated the nutrition outcome as a function of the fixed effect with a random intercept effect of the post nested within the celebrity. In addition, separate models assessed whether followers liked or commented on posts as a function of a post’s nutrition score with a random effect of the celebrity. Likes and comments had skewed distributions for foods and beverages (skews >3.2) and were log transformed to achieve normal distributions (transformed skews <0.8). Two-sided *P* <.05 was considered statistically significant.

## Results

### Celebrity Characteristics

The sample of 181 celebrities included 66 actors, actresses, and television personalities (36.5%); 64 music artists (35.4%); and 51 athletes (28.2%). A total of 102 celebrities (56.4%) were male and 79 (43.6%) were female, with a median age of 32 years (range, 17-73 years) (eTable 1 in the Supplement). At the time of data collection, these 181 celebrities had 5.7 billion total followers.^40^

### Food and Beverage Categories

The sample comprised 3065 social media posts containing 5180 total foods and beverages (2467 foods [47.6%] and 2713 beverages [52.4%]). Among foods (Table 1), snacks and sweets were 3 times more common than any other category (920 [37.3%]), followed by fruits (313 [12.7%]), proteins (295 [12.0%]), mixed dishes (271 [11.0%]), vegetables (269 [10.9%]), and grains (227 [9.2%]). Among beverages (Table 2), half were alcoholic beverages (1375 [50.7%]), followed by coffee and tea (524 [19.3%]), sweetened beverages (374 [13.8%]), and water (328 [12.1%]).

**Table 1.** Food Categories Depicted in Celebrity Social Media Posts

Food category^**a**^	Foods, No. (%) (N = 2467)
Snacks and sweets	
All	920 (37.3)
Sweet bakery products (cookies, pies, pastries, cakes, donuts, and brownies)	524 (21.2)
Candy (candy, chocolate, and caramels)	160 (6.5)
Savory snacks (cheese balls, pretzels, potato chips, popcorn, and tortilla chips)	111 (4.5)
Other desserts (ice cream and frozen dairy desserts, puddings, and gelatins)	96 (3.9)
Crackers	15 (0.6)
Snack or meal bars (breakfast bars, energy bars, and granola bars)	14 (0.6)
Fruits	313 (12.7)
Proteins	
All	295 (12.0)
Poultry (chicken, turkey, and duck)	73 (3.0)
Meats (pork, lamb, beef, goat, and game)	55 (2.2)
Seafood (fish, shellfish)	51 (2.1)
Cured meats or poultry (cold cuts, bacon, sausages, and hot dogs)	40 (1.6)
Eggs (including omelets)	40 (1.6)
Plant-based proteins (nuts, seeds, soy products, beans, and legumes)	36 (1.5)
Mixed dishes	
All	271 (11.0)
Sandwiches (cheeseburgers, deli subs, hot dogs, and peanut butter and jelly)	84 (3.4)
Grain-based (lasagna, macaroni and cheese, pasta, and rice dishes)	69 (2.8)
Pizza	37 (1.5)
Soups	23 (0.9)
Asian (chow mein, stir-fry, egg rolls, dumplings, and sushi)	21 (0.9)
Meat, poultry, and seafood	20 (0.8)
Mexican (burritos, tacos, and nachos)	17 (0.7)
Vegetables	
All	269 (10.9)
Vegetables (dark green, starchy, red or orange, leafy salads, and mixed vegetable dishes)	200 (8.1)
White potatoes (mashed, baked, fried, boiled, and French fries)	69 (2.8)
Grains	
All	227 (9.2)
Breads, rolls, tortillas (bread loaves, buns, dinner rolls, tortillas, and bagels)	98 (4.0)
Quick breads or bread products (biscuits, muffins, pancakes, and waffles)	64 (2.6)
Cereals (ready to eat)	34 (1.4)
Cooked grains (dry or plain pasta, noodles, and rice)	25 (1.0)
Cooked cereals (oatmeal, breakfast grits)	6 (0.2)
Condiments and sauces (ketchup, mustard, soy sauce, dips, gravy, and sauces)	62 (2.5)
Dairy	
All	43 (1.7)
Cheese	31 (1.3)
Yogurt	12 (0.5)
Fats and oils (butter, cream cheese, whipped cream, mayonnaise, and vegetable oils)	39 (1.6)
Sugars (sugar, honey, sugar substitutes, jams, syrups, and toppings)	21 (0.9)
Other (protein and nutritional powders)	7 (0.3)

^a^ Food categories and subcategories were defined based on What We Eat in America categories, 2015-2016.^50^

**Table 2.** Beverage Categories Depicted in Celebrity Social Media Posts

Beverage category^**a**^	Beverages, No. (%) (N = 2713)
Alcoholic beverages	
All	1375 (50.7)
Wine	597 (22.0)
Liquor and cocktails	504 (18.6)
Beer	274 (10.1)
Coffee and tea	
All	524 (19.3)
Coffee (coffee, cappuccino, blended coffee drinks, and mocha)	396 (14.6)
Tea (tea, sweet tea)	128 (4.7)
Sweetened beverages	
All	374 (13.8)
Soft drinks	224 (8.3)
Sport and energy drinks	69 (2.5)
Fruit drinks	45 (1.7)
Smoothies and grain drinks	29 (1.1)
Nutritional beverages	7 (0.3)
Water	
All	328 (12.1)
Plain water	282 (10.4)
Flavored or enhanced water	46 (1.7)
100% Juices	
All	70 (2.6)
Citrus juice	31 (1.1)
Other fruit juice	31 (1.1)
Apple juice	8 (0.3)
Vegetable juice	0
Dairy beverages	
All	37 (1.4)
Milk	15 (0.6)
Milkshakes and other dairy drinks	13 (0.5)
Flavored milk	5 (0.2)
Milk substitutes (almond, soy)	4 (0.1)
Diet beverages	
All	5 (0.2)
Diet soft drinks	4 (0.1)
Diet sport and energy drinks	1
Other diet drinks	0
Infant formula or human milk	0

^a^ Beverage categories and subcategories were defined by What We Eat in America categories, 2015-2016.^50^

### Nutrition Quality Ratings

**Figure 1.**
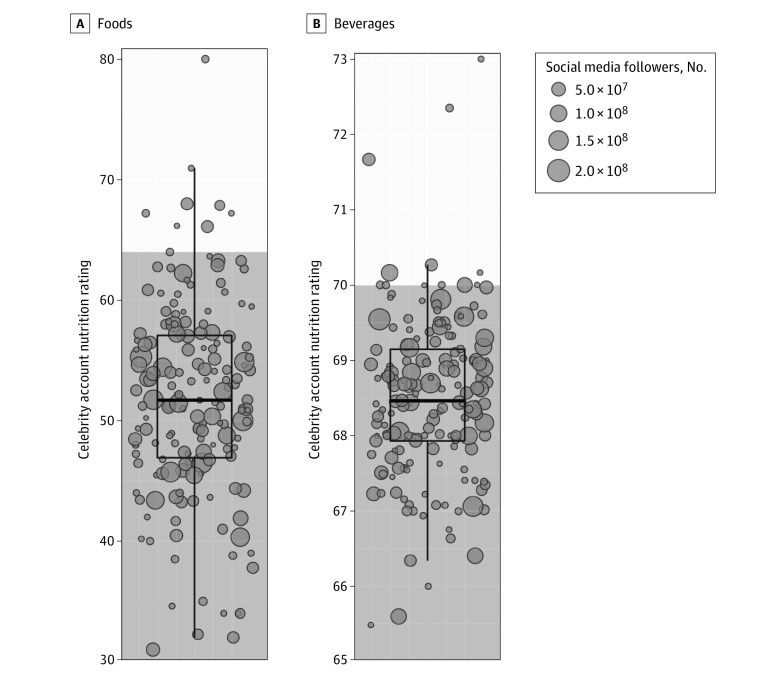
Nutrient Profile Index Nutrition Ratings of Celebrity Social Media Accounts Unshaded regions represent nutrition scores classified as “healthier,” and gray shaded regions represent nutrition scores classified as less healthy by the Nutrition Profile Index. Boxes represent the IQRs (25th and 75th percentiles), the inner horizontal line of each box represents the median, and vertical whiskers represent 1.5 times the IQR. Each dot represents the mean Nutrition Profile Index nutrition score for 1 celebrity, and dot size corresponds to the number of social media followers, with larger size indicating more followers.

Results showed that 158 of 181 celebrity social media accounts (87.3%) received a less healthy overall food nutrition score (Figure 1), which would be unhealthy enough to fail legal youth advertising limits in the UK.^7^ Nine celebrities (5.0%) received a healthier food nutrition score, and 14 celebrities (7.7%) did not depict any foods. For beverages, 162 of 181 celebrity social media accounts (89.5%) received a less healthy overall beverage nutrition score, 12 (6.6%) received a healthier nutrition score, and 7 (3.9%) did not depict any beverages. At the level of individual food and beverage items (eTable 3 in the Supplement), 1493 of 2467 foods (60.5%) and 1488 of 2713 beverages (54.8%) received less healthy nutrition scores.

Front-of-package traffic light ratings for foods (eFigure 1 and eTable 3 in the Supplement) showed that celebrity social media account nutrition scores tended to be less healthy owing to sugar and saturated fat content than to sodium content. Most celebrity accounts depicted foods with medium or high overall levels (amber or red traffic light) of sugar (153 celebrities [84.5%]), saturated fat (157 [86.7%]), and total fat (166 [91.7%]) and, to a lesser degree, sodium (114 [63.0%]). Among the 2467 food items, medium or high nutrient levels were depicted in 1339 foods (54.3%) for sugar, 1478 (59.9%) for saturated fat, 1688 (68.4%) for total fat, and 1360 (55.1%) for sodium.

### Trends by Celebrity Profession

For foods, there were no significant differences in NPI nutrition scores of posts among the 3 celebrity professions of athletes, music artists, and actors, actresses, and television personalities (eTable 4 in the Supplement). For beverages, music artists posted significantly less healthy beverages than did actors, actresses, and television personalities (*b*, −0.46; 95% CI, −0.81 to −0.10; *P* = .01) and athletes (*b*, −0.66; 95% CI, −1.03 to −0.29; *P* < .001) (eFigure 2 in the Supplement). This difference was primarily attributable to significantly higher sugar content in grams per 100 g of beverage in posts by music artists compared with posts by actors, actresses, and television personalities (*b*, 0.92; 95% CI, 0.36-1.48; *P* = .002) and athletes (*b*, 0.62; 95% CI, 0.03-1.21; *P* = .04). Alcoholic content of beverages in grams per 100 g of beverage did not differ by celebrity profession.

### Trends by Celebrity Gender

Compared with females, males did not post significantly less healthy foods (*b*, 0.84; 95% CI, −1.35 to 3.03; *P* = .45) or beverages (*b*, −0.23; 95% CI, −0.54 to 0.08; *P* = .15) (eTable 4 in the Supplement) overall. However, female celebrities posted foods with higher sugar content in grams than did males (*b*, −3.54; 95% CI, −5.83 to −1.25; *P* = .003), whereas males posted foods with higher sodium content in milligrams (*b*, 33.5; 95% CI, 4.1-62.8; *P* = .03) and lower fiber content in grams (*b*, −0.25; 95% CI, −0.47 to −0.03; *P* = .03) than did females (eTable 4 in the Supplement). Males also posted beverages with higher alcohol content in grams than did females (*b*, 1.27; 95% CI, 0.05-2.48; *P* = .04) (eFigure 2 in the Supplement).

### Social Media Posts Sponsored by Food and Beverage Companies

Only 147 of the 3065 food- or beverage-containing posts (4.8%) were explicitly indicated as sponsored by a food- or beverage-relevant company (eTable 5 in the Supplement). The 82 foods in sponsored posts (eTable 6 in the Supplement) were not rated as significantly less healthy than foods in nonsponsored posts (*b*, −1.25; 95% CI, −6.26 to 3.77; *P* = .63). However, the 142 beverages in sponsored posts were rated as significantly less healthy than beverages in nonsponsored posts (*b*, −0.59; 95% CI, −1.11 to −0.07; *P* = .03). Nearly 2 in 3 beverages (90 of 142 [63.4%]) in sponsored posts were alcoholic beverages (eTable 7 in the Supplement), and alcohol content was more than twice as high in beverages depicted in sponsored vs nonsponsored posts (10.8 g [95% CI, 9.3 g to 12.3 g] per 100 g of beverage vs 5.3 g [95% CI, 4.7 g to 5.9 g] per 100 g of beverage; difference, 5.5 g; 95% CI, 4.0 g to 7.0 g; *P* < .001) (eFigure 2 in the Supplement).

### Follower Interactions: Likes and Comments

**Figure 2.**
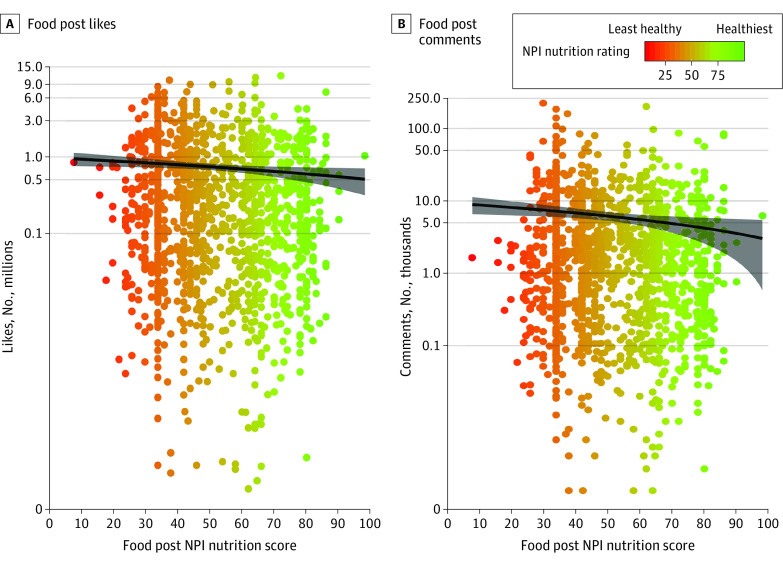
Association of Social Media Food Post Healthiness With Likes and Comments From Followers Likes and comments from social media followers per post are shown as a function of the food post Nutrition Profile Index (NPI) nutrition scores. Each dot represents 1 social media post depicting food, and the dot color represents the nutrition score of the post (green, healthiest; red, least healthy). Regression lines are fitted (gray shading indicates 95% CIs). Data are plotted on a log scale.

For foods, posts with healthier nutrition scores were associated with significantly fewer likes (*b, *−0.003; 95% CI, −0.006 to 0.000; *P* = .04) and significantly fewer comments (*b*, −0.006; 95% CI, −0.009 to −0.003; *P* < .001; Figure 2). For beverages, nutrition scores were not significantly associated with follower likes (*b*, −0.010; 95% CI, −0.025 to 0.005; *P* = .18) or comments (*b*, −0.003; 95% CI, −0.022 to 0.016; *P* = .73).

## Discussion

In this cross-sectional study, sweet bakery products and alcoholic beverages were the most commonly depicted foods and beverages in social media posts of highly followed celebrities. The overall nutrition score for more than 87% of celebrity social media accounts in this sample would be unhealthy enough to fail legal youth advertising standards in the UK. Posting such foods and beverages can shape followers’ perceptions of what is normative to consume.^16,17,18^ Moreover, food posts with less healthy nutrition scores were associated with increased likes and comments from followers, indicating greater social approval.^15^

The profile of beverages was noteworthy. Half of all beverages depicted on celebrity social media accounts were alcoholic beverages, as were nearly two-thirds (63.4%) of beverages in sponsored posts. This finding is consistent with research on the ease with which youths can access alcohol content on social media^16,17,18,26,28^ and with the finding that social media posts frequently associate alcohol with positive attributes.^28^ Depicting alcohol as such a large share of beverages matters because social media exposure to alcohol content is associated with alcohol consumption in adolescents and young adults,^17,58^ and these processes are mediated, in part, by perceived norms.^16,17,18^ Although beverage nutrition scores were not associated with likes and comments, trends by celebrity demographics suggested that beverage posts by male celebrities depicted higher alcohol content than did beverage posts by female celebrities. In addition, beverage posts by music artists depicted higher sugar content than did beverage posts from athletes or from actors, actresses, and television personalities.

Most (95.2%) of the celebrity social media posts depicting foods and beverages in this sample were not sponsored by food and beverage companies. They were primarily nonsponsored depictions of the role of foods and beverages in celebrities’ everyday lives. These results suggest that influential depictions of consumption of unhealthy foods and beverages on social media are a sociocultural problem that extends beyond advertisements and sponsorships. Celebrities are, of course, entitled to post foods and beverages as they wish on their personal social media. They themselves are individuals existing in societies that value and normalize unhealthy consumption, and it is possible that social media posts by the general public are similarly unhealthy. An association between unhealthy food posts and increased follower engagement, as found in this study, provides a potential incentive to post about unhealthy foods. However, given celebrities’ broad following, there is potential to shape their followers’ perceptions that healthy eating is normative and valued if celebrities commit to posting a healthier profile of foods and beverages. It is also important for followers to remember that social media likely represents a curated, incomplete window into what celebrities actually consume.

### Limitations

This study has several limitations. We restricted our analyses to social media photos, but videos are also common and contain engaging content. Foods and beverages were quantified as distinct instances rather than portion sizes for comparisons of nutritional quality, but portion sizes also communicate consumption norms.^59^ Rather than collecting nutrition information from various sources, we chose to obtain all nutritional information from the FNDDS. However, some brands did not have a specific FNDDS entry, and therefore, nutrition information for a more general form of that food was used (eg, “cheeseburger, not further specified”). In our estimation, White celebrities may have been overrepresented, and those from Asian, Latinx, Native American, and other racial and ethnic groups may have been underrepresented, perhaps in part owing to biases on the published lists from which we drew the celebrity sample. We could not locate public racial and ethnic identity statements for many of the celebrities, preventing reliable subgroup analyses by race and ethnicity. In addition, we chose to focus on traditional celebrities rather than social media influencers because celebrities compose a majority of the most followed social media accounts,^40^ and doing so allowed for comparison of this study’s findings with the literature on celebrity endorsements. Additional research is needed to understand whether celebrities and social media influencers differentially affect followers’ health behaviors. We did not explore effects on followers’ eating and drinking behavior.

## Conclusions

Celebrities have long been sponsored by primarily unhealthy foods and beverages in traditional advertisements. The findings of this cross-sectional study advance understanding of the nutritional quality of foods and beverages, sponsored and unsponsored, that celebrities post on their personal social media. Among 5180 foods and beverages from 181 highly followed celebrities, more than 87% of celebrity social media accounts depicted less healthy overall nutritional content. Posts were dominated by nonsponsored depictions of foods and beverages in celebrities’ everyday lives as opposed to sponsored ads from food and beverage companies. Given celebrities’ role model status and broad reach, improvements in the nutritional quality of their social media posts may be a potential opportunity to change the profile of foods and beverages that are perceived as normative and desirable to consume.

## Notes

Supplement. **eMethods.** Supplementary Methods **eTable 1.** Celebrity Demographic Characteristics **eTable 2.** Detailed Coding Decisions **eTable 3.** Celebrity-Level and Item-Level Nutrition Ratings for Foods and Beverages **eTable 4.** Food Nutrition Outcomes by Celebrity and Post Characteristics **eTable 5.** Sponsored Celebrity Instagram Posts **eTable 6.** Foods in Sponsored Celebrity Instagram Posts **eTable 7.** Beverages in Sponsored Celebrity Instagram Posts **eFigure 1.** Front-of-Package Traffic Light Ratings for Foods, by Celebrity **eFigure 2.** Beverage Nutrition Outcomes by Celebrity and Post Characteristics
eReferences
