# Supplementary material for: Nutritional Analysis of Foods and Beverages Posted in Social Media Accounts of Highly Followed Celebrities
Source: JAMA Netw Open. 2022 Jan 12;5(1):e2143087. doi: 10.1001/jamanetworkopen.2021.43087 (PMC8756336; doi:10.1001/jamanetworkopen.2021.43087)
Supplement: Supplement. — eMethods. Supplementary Methods eTable 1. Celebrity Demographic Characteristics eTable 2. Detailed Coding Decisions eTable 3. Celebrity-Level and Item-Level Nutrition Ratings for Foods and Beverages eTable 4. Food Nutrition Outcomes by Celebrity and Post Characteristics eTable 5. Sponsored Celebrity Instagram Posts eTable 6. Foods in Sponsored Celebrity Instagram Posts eTable 7. Beverages in Sponsored Celebrity Instagram Posts eFigure 1. Front-of-Package Traffic Light Ratings for Foods, by Celebrity eFigure 2. Beverage Nutrition Outcomes by Celebrity and Post Characteristics eReferences [file jamanetwopen-e2143087-s001.pdf]

Supplementary Online Content

Turnwald BP, Anderson KG, Markus HR, Crum AJ. Nutritional analysis of foods and beverages posted in social media accounts of highly followed celebrities. *JAMA Netw Open*. 2022;5(1):e2143087. doi:10.1001/jamanetworkopen.2021.43087

eMethods. Supplementary Methods

eTable 1. Celebrity Demographic Characteristics

eTable 2. Detailed Coding Decisions

eTable 3. Celebrity-Level and Item-Level Nutrition Ratings for Foods and Beverages

eTable 4. Food Nutrition Outcomes by Celebrity and Post Characteristics

eTable 5. Sponsored Celebrity Instagram Posts

eTable 6. Foods in Sponsored Celebrity Instagram Posts

eTable 7. Beverages in Sponsored Celebrity Instagram Posts

eFigure 1. Front-of-Package Traffic Light Ratings for Foods, by Celebrity

eFigure 2. Beverage Nutrition Outcomes by Celebrity and Post Characteristics

eReferences

This supplementary material has been provided by the authors to give readers additional information about their work.

© 2022 Turnwald BP et al. *JAMA Network Open*.

eMethods. Supplementary Methods

Coder reliability. Inter-rater reliability between the two researchers who conducted the coding (the first and second authors) was rigorously monitored at each step. First, to check agreement on the types of foods and beverages depicted in celebrity Instagram posts, both researchers coded which foods and beverages were present in each post for the same random sample of 307 posts (10% of the entire sample), which contained 262 foods and 235 beverages. Codes agreed perfectly in 86.0% of these posts (264 out of 307). Among individual foods and beverage observations, inter-rater reliabilities for agreeing on the type of food and type of beverage present ranged from .80 – .96 for food categories and .85 – .94 for beverage categories that had at least five observations. Second, we determined whether researchers assigned consistent nutritional information to each food and beverage observation. Both researchers assigned the closest matching nutrition code from the Food and Nutrient Database for Dietary Studies (FNDDS)[1] to a random 25% sample of all unique food and beverage items in the data set ($N = 301$). Codes agreed on 92.7% of foods and 96.4% of beverages, indicating high agreement. Third, we determined reliability of coding whether or not a post was sponsored by a food- or beverage-relevant company. Using the same set of 307 posts used to calculate reliability for the types of food and beverages depicted, codes agreed on 96.7% of posts ($\kappa = .82$). Finally, we determined reliability of coding the gender that celebrities presented themselves as, for which coders had 100% agreement. In all cases, disagreements were settled by discussion and the remainder of the data were independently coded.

© 2022 Turnwald BP et al. *JAMA Network Open*.

eTable 1. Celebrity Demographic Characteristics

	**Actor/ TV Personality** (*N* = 66)		**Music Artist** (*N* = 64)		**Athlete** (*N* = 51)		**All Celebrities** (*N* = 181)	
Gender								
Male	28	42.4%	34	53.1%	40	78.4%	102	56.4%
Female	38	57.6%	30	46.9%	11	21.6%	79	43.6%
Age								
17-29	13	19.7%	33	51.6%	11	21.6%	57	31.5%
30-39	24	36.4%	18	28.1%	34	66.7%	76	42.0%
40-49	18	27.3%	8	12.5%	6	11.8%	32	17.7%
50+	11	16.7%	5	7.8%	0	0.0%	16	8.8%

© 2022 Turnwald BP et al. *JAMA Network Open*.

eTable 2. Detailed Coding Decisions

Situation Description	Coding Decision
Pumpkins that are carved jack-o-lanterns	Excluded
Food and beverage costumes	Excluded
Medicines, cough drops, vitamins	Excluded
Salt, pepper, herbs, spices	Excluded because FNDDS does not categorize these foods
Generic words on storefronts (e.g., “pizza”, “coffee”)	Excluded
Foods within beverages (e.g., olives, lemons, limes, cherries)	Excluded
Text describing foods or beverages that are not visible at all	Excluded
Chain/branded storefronts depicted but no foods or beverages (e.g., McDonald’s arches, Burger King sign)	Excluded
Extra toppings such as on cakes, pizza, desserts, sandwiches	Excluded, toppings did not count as separate items but were sometimes included in relevant FNDDS code
Foods or beverages that cannot be identified by the image alone, but are explicitly stated in the post caption	Included
Empty boxes, wrappers, cans, bottles	Included
Foods and beverages on signs, posters, t-shirts, paintings	Included
Opaque coffee mugs containing a hot beverage	Included, counted as coffee
Beverages in champagne glasses	Included, counted as champagne
Salad with unknown salad dressing	Included, used FNDDS code for general salad dressing
Beverages known to be alcoholic from post context but type of alcohol not discernable (e.g., solo cups, opaque bottle)	Included, coded as “Alcoholic beverage, not further specified”

© 2022 Turnwald BP et al. *JAMA Network Open*.

eTable 3. Celebrity-Level and Item-Level Nutrition Ratings for Foods and Beverages

	Celebrity Food Scores		All Food Items		Celebrity Beverage Scores[a]		All Beverage Items	
	No.	%	No.	%	No.	%	No.	%
**NPI ratings**								
Healthier	9	5.0	974	39.5	12	6.6	1,225	45.2
Less Healthy	158	87.3	1,493	60.5	162	89.5	1,488	54.8
No food/bev posts	14	7.7			7	3.9		
**Traffic light ratings**								
Sugar								
low	14	7.7	1,128	45.7				
medium	110	60.8	623	25.3				
high	43	23.8	716	29.0				
no food posts	14	7.7						
Saturated fat								
low	10	5.5	989	40.1				
medium	113	62.4	669	27.1				
high	44	24.3	809	32.8				
no food posts	14	7.7						
Total fat								
low	1	0.6	779	31.6				
medium	143	79.0	894	36.2				
high	23	12.7	794	32.2				
no food posts	14	7.7						
Sodium								
low	53	29.3	1,107	44.9				
medium	114	63.0	1,311	53.1				
high	0	0.0	49	2.0				
no food posts	14	7.7						

[a]Because few beverages contain fat or sodium, traffic light ratings were not calculated for beverages.

NPI = Nutrient Profile Index

© 2022 Turnwald BP et al. *JAMA Network Open*.

eTable 4. Food Nutrition Outcomes by Celebrity and Post Characteristics

	NPI score	Sugar (g)	Sat. fat (g)	Total fat (g)	Sodium (mg)	Fiber (g)
Gender						
Male	53.0 (51.4, 54.5)	15.8 (14.2, 17.4)[a]	4.0 (3.6, 4.4)	12.1 (11.3, 13.0)	355 (335, 375)[a]	1.9 (1.7, 2.0)[a]
Female	52.1 (50.5, 53.7)	19.3 (17.7, 21.0)[a]	4.0 (3.6, 4.4)	12.4 (11.5, 13.2)	321 (300, 343)[a]	2.1 (2.0, 2.3)[a]
Profession						
Actor	53.2 (51.4, 54.9)	17.0 (15.1, 18.9)	4.0 (3.6, 4.4)	12.1 (11.2, 13.1)	329 (305, 353)	2.0 (1.8, 2.2)
Music Artist	50.9 (48.8, 52.9)	18.9 (16.8, 21.1)	4.0 (3.5, 4.5)	12.8 (11.6, 13.9)	357 (328, 385)	2.1 (1.9, 2.3)
Athlete	53.4 (51.5, 55.3)	16.7 (14.6, 18.7)	4.0 (3.5, 4.4)	11.9 (10.9, 13.0)	336 (310, 362)	1.9 (1.7, 2.1)
Sponsored						
Sponsored	51.4 (46.4, 56.3)	20.1 (15.0, 25.3)	3.4 (2.2, 4.6)	11.5 (8.6, 14.4)	364 (287, 439)	2.1 (1.6, 2.6)
Not Sponsored	52.6 (51.5, 53.7)	17.4 (16.2, 18.6)	4.0 (3.8, 4.3)	12.3 (11.7, 12.9)	338 (323, 353)	2.0 (1.9, 2.1)

[a] Rows within column differed significantly from one another ($P < .05$)

eTable 5. Sponsored Celebrity Instagram Posts

Sponsor or Product Name	Sponsored posts in this sample
Proper Twelve Irish Whiskey	16
Smithworks vodka	8
Vitamin Well	7
Body Armor	6
Sugarpova candy	6
Mulholland Distilling	6
Wade Cellars	5
Ronaldinho Gaucho wine	5
Iniesta	5
Codigo tequila	5
Bud Light beer	5
Aviation American gin	5
Blanco tequila	4
State Energy water	3
Jacob’s Creek wine	3
Epigamia	3
Red Bull	3
Outlaw energy drink	2
Salt Vine Wines	2
Chobani	2
Cheetos	2
Lay’s chips	2
Ultra Light beer	2
Haig Club	2
Heinz ketchup	2
Bragg apple cider vinegar	2
Poosh	2
Herbalife nutrition	2
Starkist	2
TB12	2
Cocoa Pebbles	2
800 degrees kitchen	1
Combat Hunger	1
Mod champagne	1
Mom’s Spaghetti	1
Pizza My Heart	1
Pepsi	1
Wheaties	1
Laird	1
Bai	1
Vita Hustle	1
Flatotummy	1
Usana	1
Vrauu	1
Malaysia Travel	1
Corona Light	1
Pestana CR7 Lisboa	1
See’s candy	1
Olive Oils Spain	1
Raising Canes Chicken	1
Miyoko Creamery	1
The Ellen Shop	1
Last Call Beverage	1

Rebbl	1
Truth bar	1

eTable 6. Foods in Sponsored Celebrity Instagram Posts

Food Category	No.	%
**Snacks and Sweets**	**24**	**29.3**
Candy (candy, chocolate, caramels)	11	13.4
Snack / Meal bars (breakfast bar, energy bar, granola bar)	7	8.5
Savory snacks (cheese balls, pretzels, potato chips, popcorn, tortilla chips)	5	6.1
Crackers	1	1.2
Sweet bakery products (cookies, pies, pastries, cakes, donuts, brownies)	0	0.0
Other desserts (ice cream and frozen dairy desserts, puddings, gelatins)	0	0.0
**Fruits**	**10**	**12.2**
**Grains**	**9**	**11.0**
Cereals (ready-to-eat)	7	8.5
Breads, rolls, tortillas (bread loaves, buns, dinner rolls, tortillas, bagels)	2	2.4
Quick breads / bread products (biscuits, muffins, pancakes, waffles)	0	0.0
Cooked grains (dry or plan pasta, noodles, rice)	0	0.0
Cooked cereals (oatmeal, breakfast grits)	0	0.0
**Dairy**	**9**	**11.0**
Yoghurt	5	6.1
Cheese	4	4.9
**Mixed Dishes**	**7**	**8.5**
Mixed Dishes – Meat, Poultry, Seafood	2	2.4
Mixed Dishes – Sandwiches (cheeseburger, deli subs, hot dogs, PBJ)	1	1.2
Mixed Dishes – Grain-based (lasagna, mac and cheese, pasta, rice dishes)	1	1.2
Mixed Dishes – Pizza	1	1.2
Mixed Dishes – Soups	1	1.2
Mixed Dishes – Mexican (burritos, tacos, nachos)	1	1.2
Mixed Dishes – Asian (chow mein, stir-fry, egg rolls, dumplings, sushi)	0	0.0
**Protein**	**6**	**7.3**
Seafood (fish, shellfish)	3	3.7
Poultry (chicken, turkey, duck)	1	1.2
Meats (pork, lamb, beef, goat, game)	1	1.2
Plant-based proteins (nuts, seeds, soy products, beans, legumes)	1	1.2
Cured Meats/Poultry (cold cuts, bacon, sausages, hot dogs)	0	0.0
Eggs (including omelets)	0	0.0
**Other (protein and nutritional powders)**	**6**	**7.3**
**Condiments and Sauces** (ketchup, mustard, soy sauce, dips, gravy, sauces)	**5**	**6.1**
**Sugars** (sugar, honey, sugar substitutes, jams, syrups, toppings)	**3**	**3.7**
**Fats and Oils** (butter, cream cheese, whipped cream, mayo, vegetable oils)	**2**	**2.4**
**Vegetables**	**1**	**1.2**
Vegetables (dark green, starchy, red/orange, leafy salads, veg mix dishes)	1	1.2
White potatoes (mashed, baked, fried, boiled, French fries)	0	0.0

Food categories (bolded) and subcategories (indented) were defined by WWEIA Categories 2015-2016.[2] Percentages out of 82 total foods in posts that were sponsored by food- and beverage-relevant companies.

eTable 7. Beverages in Sponsored Celebrity Instagram Posts

Beverage Category	No.	%
**Alcoholic Beverages**	**90**	**63.4**
Liquor and Cocktails	54	38.0
Wine	28	19.7
Beer	8	5.6
**Sweetened Beverages**	**25**	**17.6**
Sport and Energy drinks	17	12.0
Nutritional beverages	4	2.8
Soft drinks	3	2.1
Smoothies and grain drinks	1	0.7
Fruit drinks	0	0.0
**Water**	**17**	**12.0**
Flavored or Enhanced Water	14	9.9
Plain Water	3	2.1
**Coffee and Tea**	**6**	**4.2**
Tea (tea, sweet tea)	4	2.8
Coffee (coffee, cappuccino, blended coffee drinks, mocha)	2	1.4
**100% Juices**	**2**	**1.4**
Citrus juice	1	0.7
Other fruit juice	1	0.7
Apple juice	0	0.0
Vegetable juice	0	0.0
**Diet beverages**	**2**	**1.4**
Diet soft drinks	2	1.4
Diet sport and energy drinks	0	0.0
Other diet drinks	0	0.0
**Dairy Beverages**	**0**	**0.0**
Milk	0	0.0
Milkshakes and Other Dairy Drinks	0	0.0
Flavored Milk	0	0.0
Milk Substitutes (almond, soy)	0	0.0
**Infant formula / Human milk**	**0**	**0.0**

Beverage categories (bolded) and subcategories (indented) were defined by WWEIA Categories 2015-2016.[2] Percentages out of 142 total beverages in posts that were sponsored by food- and beverage-relevant companies.

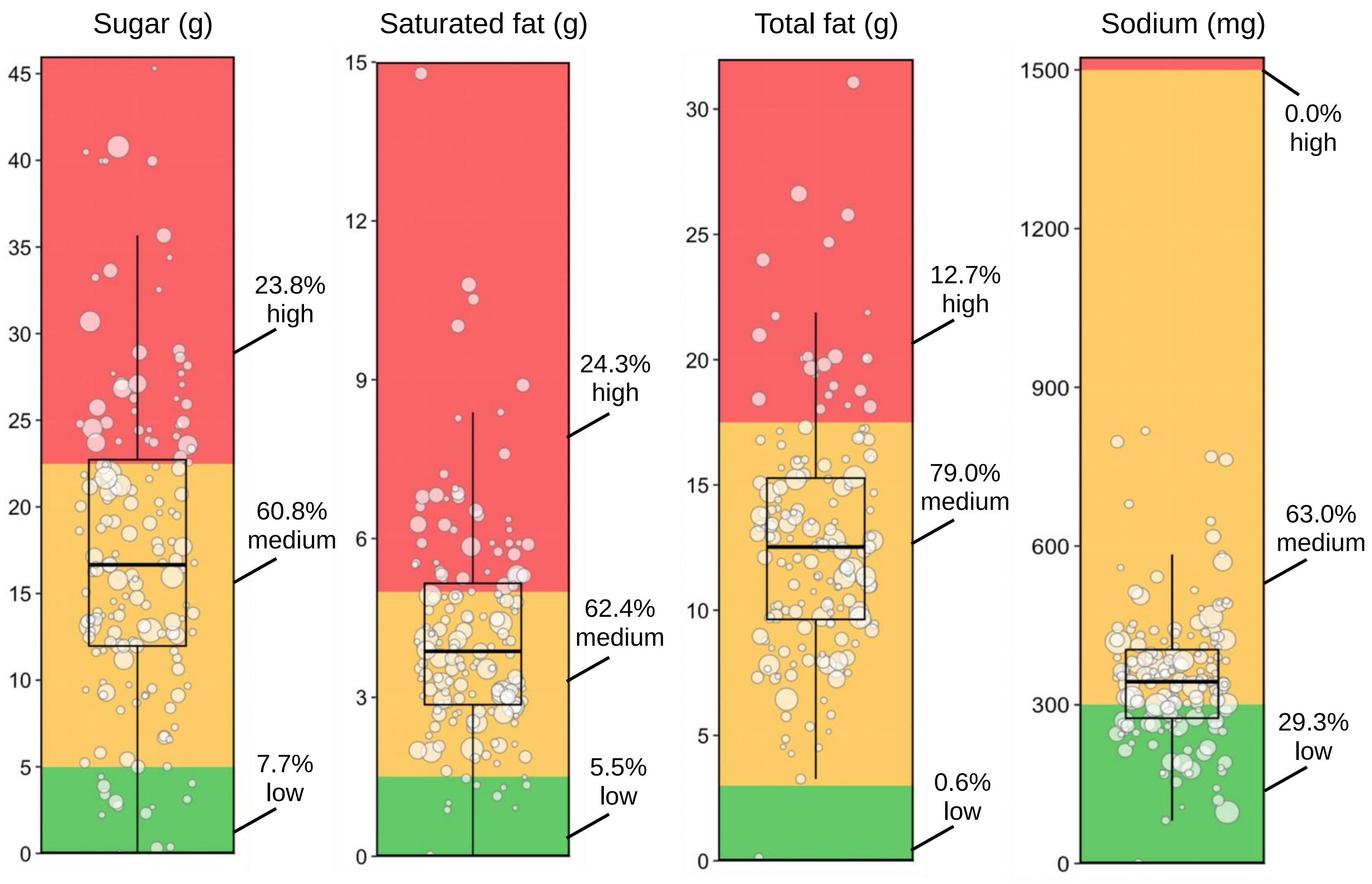

eFigure 1. Front-of-Package Traffic Light Ratings for Foods, by Celebrity.
Celebrity Instagram account-level "traffic light" nutrition ratings for foods. Each dot represents one celebrity's average sugar, saturated fat, total fat, or sodium content per 100 g of food (*n* = 167 celebrities with at least one post containing food). Percentages do not add to 100 because 7.7% (*n* = 14 celebrities) had no food-containing posts. Green shading represents low levels (healthiest), amber represents medium, and red represents high levels of each nutrient (least healthy), per Front-of-Package Traffic Light labeling guidelines.[3] Each boxplot inner horizontal line represents the median, boxes represent the interquartile range (25th and 75th percentiles), and vertical whiskers represent 1.5 times the interquartile range. Dot size corresponds to the number of Instagram followers per celebrity.

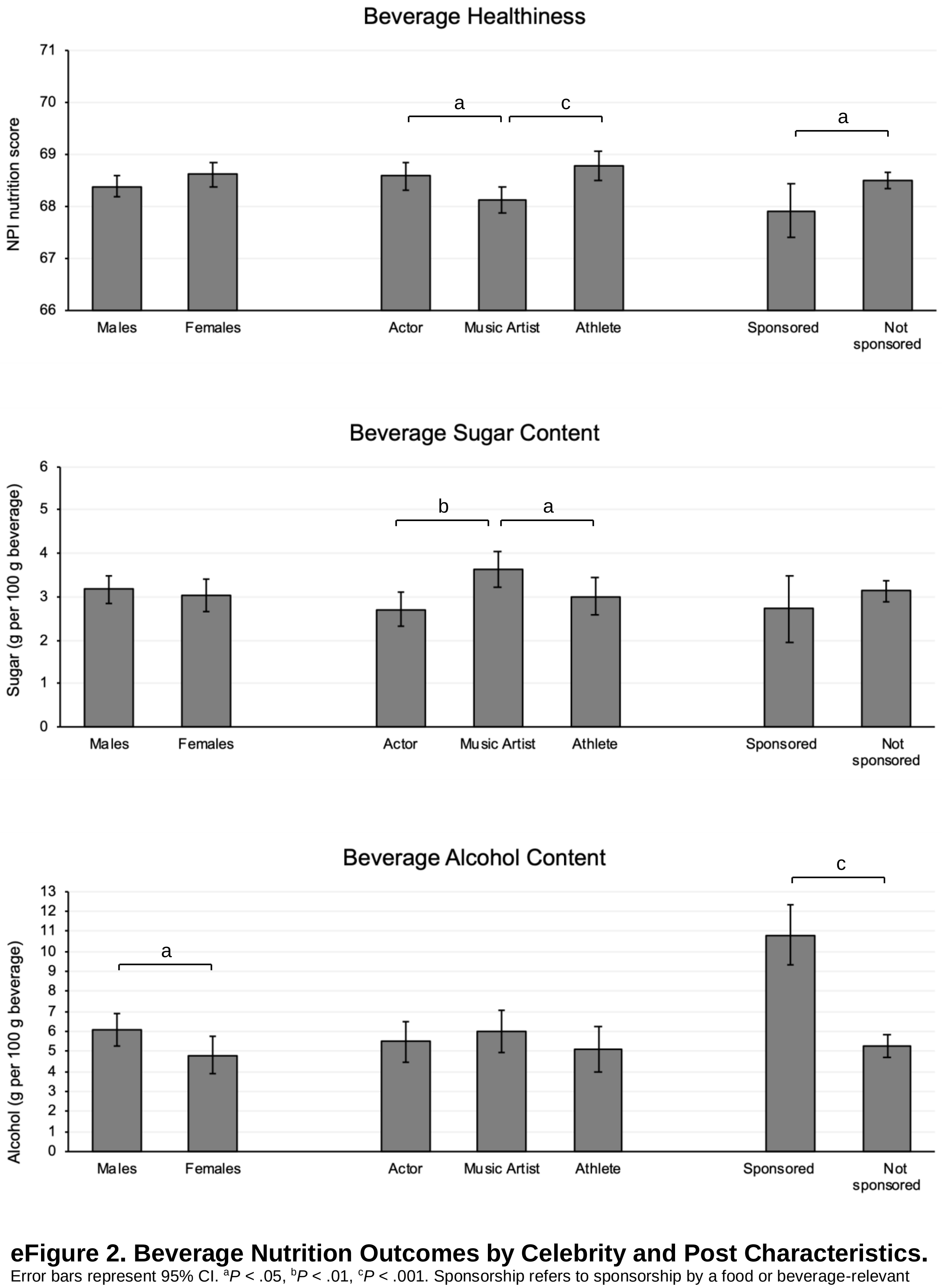

eFigure 2. Beverage Nutrition Outcomes by Celebrity and Post Characteristics.
Error bars represent 95% CI. [a]$P < .05$, [b]$P < .01$, [c]$P < .001$. Sponsorship refers to sponsorship by a food or beverage-relevant company.

© 2022 Turnwald BP et al. *JAMA Network Open*.

eReferences

1. U.S. Department of Agriculture, Agricultural Research Service. USDA Food and Nutrient Database for Dietary Studies 2015-2016. Food Surveys Research Group Home Page. Accessed February 11, 2020. http://www.ars.usda.gov/nea/bhnrc/fsrg.
2. U.S. Department of Agriculture. What We Eat in America Food Categories 2015-2016. Accessed February 11, 2020. https://www.ars.usda.gov/ARSUserFiles/80400530/pdf/1516/Food_categories_2015-2016.pdf.
3. Department of Health. Guide to creating a front of pack (FoP) nutrition label for pre-packed products sold through retail outlets. Accessed January 24, 2020. https://www.food.gov.uk/sites/default/files/media/document/fop-guidance_0.pdf.
